# GECKO is a genetic algorithm to classify and explore high throughput sequencing data

**DOI:** 10.1038/s42003-019-0456-9

**Published:** 2019-06-20

**Authors:** Aubin Thomas, Sylvain Barriere, Lucile Broseus, Julie Brooke, Claudio Lorenzi, Jean-Philippe Villemin, Gregory Beurier, Robert Sabatier, Christelle Reynes, Alban Mancheron, William Ritchie

**Affiliations:** 1 Institute of Human Genetics, CNRS UPR1142, Machine learning and gene regulation, University of Montpellier, Montpellier, France; 2 AGAP, Univ Montpellier, CIRAD, INRA, Montpellier SupAgro, Montpellier, France; 3 IGF, Centre National de la Recherche Scientifique, INSERM U1191, University of Montpellier, Montpellier, France; 4 LIRMM, Université de Montpellier, CNRS, UMR5506, Montpellier, France; 5 Institut Biologie Computationnelle, Montpellier, France

**Keywords:** Machine learning, Predictive medicine

## Abstract

Comparative analysis of high throughput sequencing data between multiple conditions often involves mapping of sequencing reads to a reference and downstream bioinformatics analyses. Both of these steps may introduce heavy bias and potential data loss. This is especially true in studies where patient transcriptomes or genomes may vary from their references, such as in cancer. Here we describe a novel approach and associated software that makes use of advances in genetic algorithms and feature selection to comprehensively explore massive volumes of sequencing data to classify and discover new sequences of interest without a mapping step and without intensive use of specialized bioinformatics pipelines. We demonstrate that our approach called GECKO for GEnetic Classification using *k-mer* Optimization is effective at classifying and extracting meaningful sequences from multiple types of sequencing approaches including mRNA, microRNA, and DNA methylome data.

## Abstract (web summary)

Aubin Thomas, Sylvain Barriere et al. present a computational method for classifying and extracting meaningful sequences from high-throughput sequencing data. The method, called GECKO, uses k-mer counts that are able to classify the input data with high accuracy.

## Introduction

Studies of variation in gene expression, initially through probe-based technology and more recently high throughput sequencing (HTS), have considerably advanced knowledge of disease etiology and classification^1–3^. The recent promotion of HTS across a wide spectrum of diseases has generated a wealth of data that measure gene expression and transcript diversity but also explore its putative genetic and epigenetic regulators. Still, despite more than a decade of development, computational analysis and integration of these data presents a major challenge. Each type of HTS experiment is compartmentalized to a set of computational pipelines and statistical approaches that often require a full-time bioinformatics specialist. In addition, most of these pipelines rely on a reference genome or transcriptome and thus cannot inherently account for the diversity in non-reference transcripts or individual variations^4^. To remove the requirement of a reference, recent methodologies use *k-mer* representation; they directly compare the counts of nucleotide sequences of length *k* between samples^5^. These approaches have been successful at detecting novel transcripts but only on a very small subset of RNA sequencing data^4^ and would be impossible to implement for the classification of large patient cohorts using the entire transcriptome. In the field of metagenomics, numerous algorithms have been developed to discover unique *k-mers* or *k-mer* signatures to classify organisms^6,7^. However, these were developed for organisms with smaller genomes that do not have billions of different *k-mers*. In addition, they were designed for inter-species studies where unique *k-mers* can be attributed to the genomes of different taxonomic identities.

Exploring a large set of *k-mers* to classify samples can be framed as a global optimization problem for which many recent approaches have been published and compared^8^. Amongst these is a class of nature-inspired algorithms termed Genetic Algorithm which are based on the processes of mutation, crossing over and natural selection. These have appealing properties that could apply to the exploration of a large set of *k-mers*. They have low memory requirements because they explore only part of the data at each stage and they can produce multiple solutions that fit well with biological interpretation of data. However, despite these properties, genetic algorithms are rarely used to optimize problems with relatively small sample sizes and such a large number of parameters, in this case billions of *k-mers*.

We have created a novel approach and associated software called GECKO for genetic classification using *k-mer* optimization that is especially designed for HTS data. GECKO is based on *k-mer* decomposition coupled with an adaptive genetic algorithm that explores HTS data from two or more input conditions. This algorithm searches for groups of *k-mers* that, combined together are highly informative; they are able to classify the input categories with high accuracy. Because GECKO uses *k-mer* counts, it can theoretically be applied to any type of HTS experiment and does not rely on a reference genome or transcriptome. Here, we successfully apply GECKO to a variety of biological problems and sequencing data. These include microRNA (miRNA) sequencing to classify normal blood cells, mRNA sequencing to classify subtypes of breast cancer and to predict response to chemotherapy, and bisulfite sequencing (BS-seq) on normal versus chronic lymphocytic leukemia (CLL) samples. Regardless of the type of data, GECKO finds small, accurate signatures that classify these samples and could thus be used as diagnostic and prognostic markers. In addition, by visualizing how the genetic algorithm evolves to find solutions, GECKO can be used to explore novel sequences or groups of functionally related sequences associated with normal biology and disease.

## Results

**Fig. 1:**
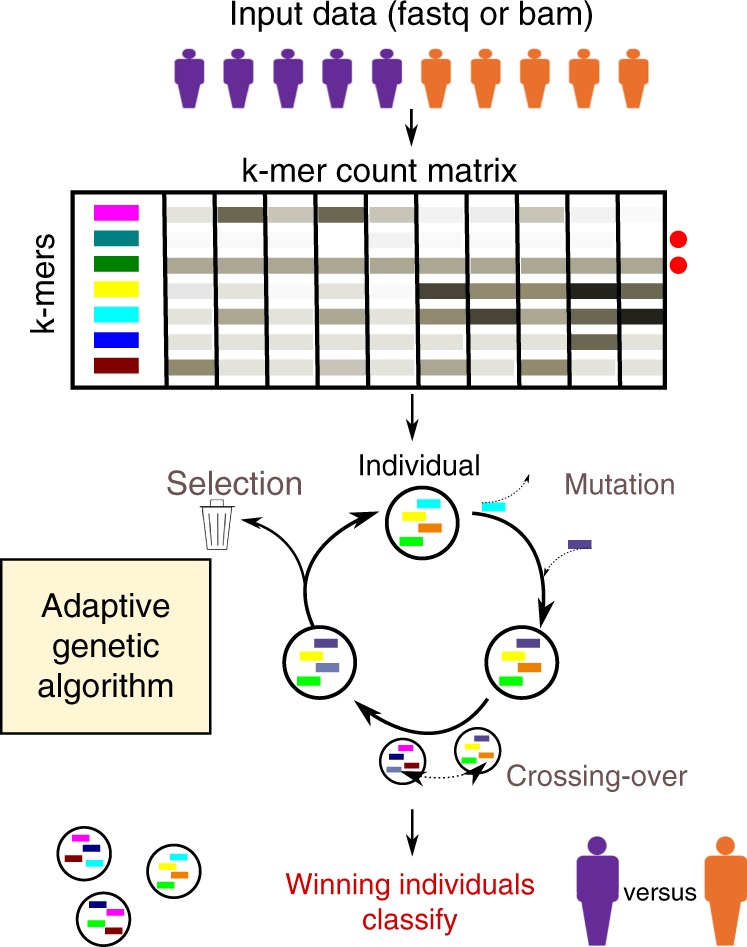
Overview of the GECKO algorithm. Input fastq or bam files from two or more conditions are transformed into a matrix of *k-mer* counts across all samples. The *k-mers* for which the counts are below a noise threshold or that do not vary across samples are removed (red dots on the right of the *k-mer* matrix). The adaptive genetic algorithm randomly selects groups of *k-mers* from the *k-mer* matrix to form individuals. These individuals will go through rounds of mutation, crossing-over and selection to discover individuals capable of classifying the input samples with high accuracy

GECKO is designed around two main steps; these are a *k-mer* matrix preparation step and an adaptive genetic algorithm (Fig. 1).

The *k-mer* matrix preparation, uses an input sequencing file (.bam or .fastq) to create a matrix of *k-mer* counts; that is the number of times a sequence of length *k* appears in each sample (*k* = 30 by default). This matrix is filtered for *k-mers* with low counts and non-informative or redundant *k-mers* (see the section “Methods”). Then, during the second step an adaptive genetic algorithm will explore the matrix to discover combinations of *k-mers* that can accurately classify input samples. The adaptive genetic algorithm starts by creating thousands of digital individuals; these are groups of randomly selected *k-mers*. The set of individuals is called a population. This population will then go through phases of mutation, where individuals replace one of their *k-mers* with another randomly selected *k-mer*; a phase of crossing-over where individuals exchange a portion of their *k-mers* with each other and selection, where individuals that do not classify the input samples well enough will be removed from the population and replaced. Mutation allows GECKO to explore local solutions similar to the individual to be mutated; crossing-over, allows GECKO to explore a broader set of solutions and reduces the chances of getting stuck in a local minimum (see the section “Methods”). Each cycle of mutation, crossing-over, and selection is called a generation. By default, GECKO will iterate through 20,000 generations or stop when the number of new solutions discovered throughout generations slows down (see stopping criteria in the section “Methods”). This algorithm is called adaptive because the mutation and crossing-over rates depend on how well individuals in the population perform. Individuals that perform well have lower rates to prevent them from changing drastically and thus enabling them to converge faster to a solution; individuals that do not perform well will have higher rates to enable wider exploration of solutions.

In the analyses presented in this study and by default in the software, GECKO’s performance is systematically tested on 1/6th of the data that is randomly selected and set aside before running the algorithm (see the section “Methods”). This test set allows us to evaluate the accuracy and overfitting for each run; it measures whether the algorithm fits too closely to the training set and thus will not correctly predict future input samples. GECKO is thus run on the remaining 5/6th of the data with cross-validation at each generation of the algorithm.

### Classifying miRNA sequencing data of blood cells

We first tested GECKO’s performance on a miRNA expression data of seven types of blood cells sorted from 43 healthy patients for a total of 413 samples^9^. We ran GECKO on this dataset using 20-mers (*k-mer* size of 20; miRNAs generally vary in size from 20 to 23) to find a set of *k-mers* that could correctly classify the seven blood-cell types.

**Fig. 2:**
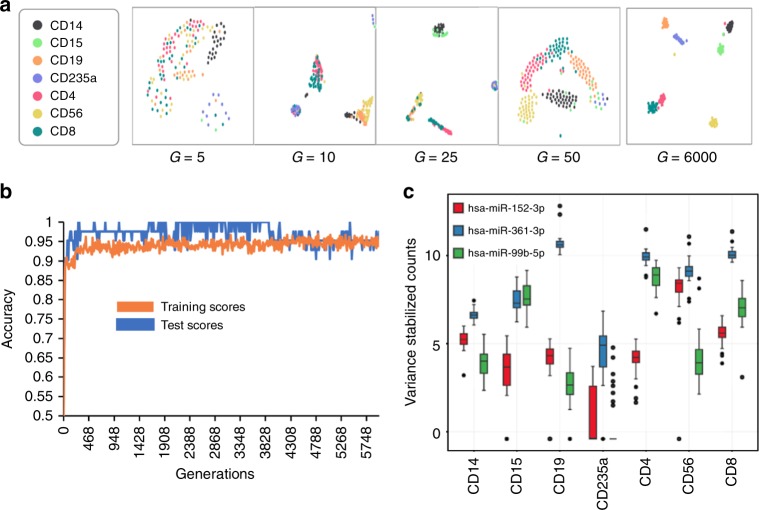
GECKO can accurately classify miRNA data from seven types of blood cells using three *k-mers*. **a** GECKO output showing the separation of the seven blood-cell types at each generation (G) of GECKO analysis using t-SNE visualization applied to *k-mer* counts. **b** GECKO output showing the accuracy of separation for the training and test set across 6000 generations. **c** variance stabilized counts of the three miRNAs that correspond to the three *k-mers* discovered by GECKO across the seven blood-cell types (*n* = 43 biologically independent donors)

After 6000 generations (15 h on 15 cores; see Supplementary Table 1 for parameters and Supplementary Fig. 1 for runtimes and memory usage) GECKO discovered an individual composed of only three *k-mers* (ACCCGTAGAACCGACCTTGC, CCCCAGGTGTGATTCTGATA, AGTGCATGACAGAACTTGGG) that could distinguish the groups with 0.96 accuracy (Fig. 2a, b and Supplementary Data 1 and 2).

In the initial study, the authors described a signature of 136 cell-type-specific miRNAs. These 136 miRNAs could classify the groups with 0.97 accuracy. Thus, we found a much smaller signature that could classify the seven blood-cell types with similar accuracy without the use of a miRNA-dedicated bioinformatics pipeline.

We then aligned the three *k-mers* discovered by GECKO to a database of known miRNAs^10^. Two of these mapped perfectly to miRNAs 152-3p and 99b-5p, which were annotated in the original study as specific to NK cells and T helper cells, respectively. The third mapped to miRNA 361-3p which was not found to be specific to any of the seven cell types and was thus ignored in the initial study. Separately, the first two *k-mers* could classify one cell-type each and the third would have been overlooked. Together these three *k-mers* classify all seven groups with high accuracy because of their contrasting expression between each cell types (Fig. 2c).

### Classifying breast cancer subtypes using mRNA sequencing data

**Fig. 3:**
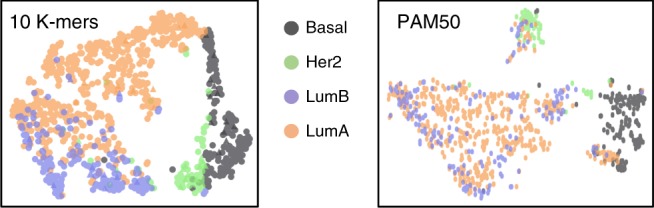
GECKO discovers 10 *30-mers* that classify breast cancer subtypes. Comparison of breast cancer subtype classification using the frequency of *k-mers* discovered by GECKO and the transcript per million values of the PAM50 gene. Panels show the t-SNE separation of the four classes

Breast cancer is a heterogeneous disease in regards to response to treatment and its transcriptional background. Defining the subtypes luminal A (LumA), luminal B (LumB), HER2-enriched (HER2) and basal-like are crucial for prognosis and predicting outcome of breast cancer. These subtypes were initially defined through unsupervised clustering of gene expression and are currently identified using a standard qPCR assay of 50 genes called the PAM50^11,12^. To assess whether GECKO could identify *k-mers* that classify breast cancer subtypes, we used a dataset of 1087 mRNA-Seq breast cancer samples from the Cancer Genome Atlas Pan-Gyn cohort^13^ (patients per class: Basal 175, Her2 73, LumA 513, LumB 185). We ran GECKO for 20,000 generations (75 h on 15 cores; see Supplementary Table 1 for parameters and Supplementary Fig. 1 for runtimes and memory usage) and extracted the highest scoring individual at its term (Supplementary Table 2). We then tested how well these *k-mers* classified the four cancer subtypes compared to PAM50 expression values calculated as transcript per million (TPM). Both the *k-mer* counts and PAM50 TPMs were trained using a linear support vector machine (see the section “Methods”) with identical training data and evaluated on the same test set. The 10 *k-mers* had higher accuracy rates compared to the PAM50 on all four classes (Fig. 3 and Table 1).

**Table 1:** Confusion matrices of breast cancer subtype classification using the frequency of *k-mers* discovered by GECKO and the transcript per million values of the PAM50 gene set

	Classification with GECKO *k-mers*						Classification with PAM50 TPM values				
Predicted class	Basal	97.7	2.2	0	0	Predicted class	Basal	86	5.2	5.5	3.3
	Her2	2	87.5	6.2	4.2		Her2	15.3	60.6	3.6	20.6
	LumA	1.5	1.5	92.3	4.6		LumA	15.3	2.2	88.1	8.6
	LumB	0	3.4	18.8	77.8		LumB	5.9	15.4	36.5	42.2
		Basal	Her2	LumA	LumB			Basal	Her2	LumA	LumB
		True class						True class			

We then further inspected the 10 *k-mers* discovered by GECKO by mapping them to the human genome. We found that four of the *k-mers* mapped to genes from the PAM50 list (FOXC1, ESR1, KRT14, KRT17). Three others mapped to genes NISCH, TPX2, and ATF3, the first of which is linked to breast cancer aggressiveness^13^ and the two latter both affect cell viability in breast cancer cells^14,15^. The three last *k-mers* mapped to three genes KLHL6, KANSL2, and PHF10 shown to be involved in tumorigenesis but not in breast cancer^16–18^. Of the 10 *k-mers*, 3 map to coding regions and 7 map to 3′ untranslated regions for which multiple isoforms exist. *k-mer* counting can thus integrate alternative transcription to classify mRNA-Seq samples.

### Classifying response to chemotherapy of triple negative breast cancer on small sample sizes of mRNA-Seq

**Fig. 4:**
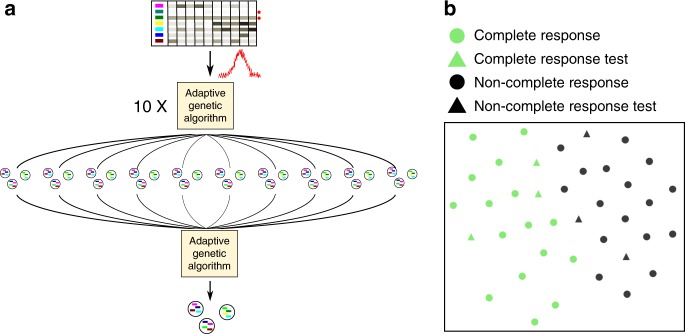
GECKO voting mode for small sample sizes. **a** GECKO’s voting mode will run 10 separate genetic algorithms with added Gaussian noise. The best solutions of these runs will be fed into a final genetic algorithm to produce a final solution. **b** GECKO output showing the t-SNE separation of patients with complete response to chemotherapy from those that did not using five *k-mers* from the winning individual. Triangles correspond to the test dataset that was excluded from GECKO training can thus be used to estimate overfitting

We then tested GECKO on a dataset with more heterogeneous cell populations and smaller sample sizes. We used a cohort of triple-negative breast cancer patients, an aggressive, heterogeneous subtype of breast cancer with poor outcomes. This cohort taken from the Breast Cancer Genome Guided Therapy (BEAUTY) study^19,20^ was divided into 19 patients that had a complete response to chemotherapy and 20 patients that did not. In such cases of small sample size and high heterogeneity, we recommend using GECKO’s voting mode (Fig. 4a).

This mode compensates for bias that may be introduced when splitting a small number of samples between training and test datasets and may thus accentuate batch effects. The voting mode will run 10 instances of the genetic algorithm for 10,000 generations. At their term, it will select *k-mers* from the top individuals across the 10 instances and run a final genetic algorithm on this subset of *k-mers* for another 10,000 generations. Running multiple genetic algorithms and aggregating their results prevents overfitting on a specific split of the data between the training and test set. In addition, the voting mode introduces Gaussian noise by default into the data to further prevent overfitting. This option is recommended for experiments with <30 samples per condition.

Using the voting mode (83 h using 15 cores; see Supplementary Table 1 for parameters and Supplementary Fig. 1 for runtimes and memory usage), we found an individual that was able to classify patients with 0.93 accuracy (Fig. 4b) with only five *k-mers* of length 30 (Supplementary Table 3). As expected three of these *k-mers* mapped to genes that had clear roles in resistance to chemotherapy; *JAK3* is involved in chemotherapy resistance in triple-negative breast cancer^8^, *BOP1* reduces chemotherapy resistance^21^ and *VTCN1* is associated with poor clinical outcomes in numerous cancers including breast cancer^22^.

### Classifying BS-seq data

We then wanted to see if GECKO could accurately classify samples using epigenetic sequencing data, such as BS-seq generated to investigate DNA methylation. BS-seq requires extensive bioinformatics processing to discover changes in methylation and thus, a method that could directly classify BS-seq samples could be of great interest. To test GECKO on BS-seq we downloaded raw sequencing files from a study on methylome diversity in 104 primary CLLs samples compared with 26 normal B cell samples^23^. Although global hypomethylation has been well described in cancer, these alterations are highly variable between CLL samples^23^ and thus present a challenge for classification.

**Fig. 5:**
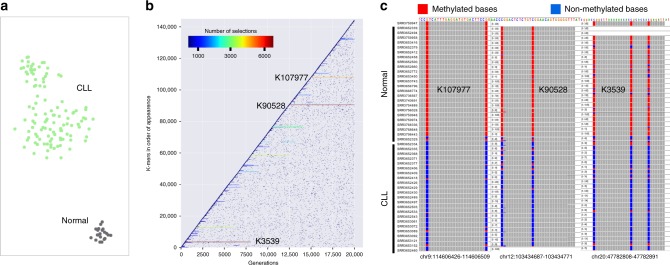
GECKO can accurately classify normal and CLL patients using *k-mers* from bisulfite sequencing data. **a** GECKO output showing the t-SNE separation of CLL and normal samples using 20 *k-mers* from the winning individual. **b** GECKO output of *K-mer* exploration across 20,000 generations; *k-mers* that are frequently found in winning organisms are displayed as horizontal lines across generations; dots represent *k-mers* that were selected in one generation but eliminated in the following generation often due to a decrease in fitness of the model. **c** IGV screenshots showing the methylation status of normal and CLL samples of regions corresponding to three most frequently used *k-mers* in winning organisms determined by the Bismark software

We ran GECKO for 20,000 generations (39 h; see Supplementary Table 1 for parameters and Supplementary Fig. 1 for runtimes and memory usage) and found a winning individual that was able to classify normal from CLL samples with an accuracy of 1 using 20 *k-mers* (Fig. 5a; Supplementary Table 4). In addition to this final classification, GECKO plots the evolution of winning organisms across the 20,000 generations (Fig. 5b). This graph can be used to identify individual *k-mers* that are essential for classification and thus worth investigating. Here we found three *k-mers* that were most frequently used by winning individuals for classification (Supplementary Table 5).

We verified the methylation status of the loci where these *k-mer* sequences were mapped using the Bismark software^24^ and found that all three of them displayed dramatic changes in DNA methylation between normal and CLL samples (Fig. 5c). Interestingly the two *k-mers* that were finally selected after 20,000 generations, K107977 and K90528 overlapped binding sites for CTCF and GATA3, both of which are affected by DNA methylation status^25,26^. K107977 overlaps a CTCF-binding site for the ATP6V1G1 gene^27^, which codes for a proton pump responsible for acidification of the cell, a hallmark of cancer promotion. K90528 overlaps a GATA3-binding site for the SULF2 gene that has already been identified as a diagnostic and prognostic marker in multiple cancers^28–30^.

## Discussion

HTS data analysis often requires extensive data transformations through tailored bioinformatics pipelines to organize the sequences in a manner that is coherent with our understanding of biology. Mapping to a reference, using ad hoc statistical thresholds and grouping sequences by functional elements, such as transcripts are common steps in most bioinformatics pipelines.

We designed GECKO with the aim of creating a classifier that could explore HTS data without a reference genome or transcriptome and without the need of bioinformatics pipelines dedicated to a specific library preparation or technology. The approach we describe here can in theory explore any type of sequencing data. Because GECKO considers groups of *k-mers* for classification, it can make use of co-dependencies between sequences to find smaller and more accurate classifiers. Thus, GECKO is capable of better classification than the commonly used approach that consists of selecting genes for which the expression is statistically significant between conditions to build a classifier (Supplementary Fig. 2). In the miRNA analysis of blood cells for example, one of the *k-mers* that participated in making an excellent classifier was not statistically significant by itself and would have been overlooked.

Using *k-mer* counts removes the requirement of a mapping step and makes GECKO applicable to numerous types of sequencing experiments. In addition, we found that using *k-mers* instead of other metrics, such as fragments per kilobase million (FPKM) or read counts resulted in higher predictive power even when run with the same genetic algorithm (Supplementary Fig. 3). This can be explained by the fact that *k-mers* can measure changes in transcription, isoform abundance, and sequence simultaneously. When applied to bisulfite converted data, each epigenetic change can potentially lead to the appearance of a novel *k-mer* in samples where the modification is present. These sample-specific *k-mers* allow GECKO to make very efficient classifications and to pinpoint the exact location of the modification.

Unlike regression analysis our approach provides multiple solutions (Supplementary Fig. 4). For research purposes this allows us to investigate why different groups of solutions work well together, explore co-dependencies between sequences and functional pathways that allow a good separation of input samples. In a clinical setting, providing multiple good solutions allows more flexibility for selecting diagnostic or prognostic targets. Importantly, the *k-mers* used for classification are not biased towards higher expressed genes (Supplementary Fig. 5) and mostly map to unique locations in the genome or transcriptome (Supplementary Fig. 6). Thus, GECKO can make use of unique transcriptional elements across a large spectrum of expression.

GECKO’s ability to work across multiple types of data without the need of dedicated bioinformatics tools could make it invaluable for cross-platform large-scale analyses but also for individual researchers and clinicians who would be able to compare HTS data between cohorts of patients with no bioinformatics training. It is worth noting that the longest and computationally intensive part of our procedure is obtaining the *k-mer* matrix. This step need be performed only once per dataset however and providing a *k-mer* matrix for online datasets along with sequencing files could result in widespread use of non-biased approaches such as GECKO. In addition, *k-mer*-based approaches, such as GECKO have the advantage of being portable; *k-mer* sequences will not change with new versions of the genome.

## Methods

### Data preparation

The *k-mer* decomposition into a matrix of *k-mer* counts is performed using Jellyfish 2^31^. This step can be preceded by a filtering of sequencing adaptors by Trim Galore (bioinformatics.babraham.ac.uk/projects/trim_galore/) if the user selects this option in GECKO. GECKO will then eliminate *k-mers* for which the count is below a noise threshold, *k-mers* that are uninformative for the given study and *k-mers* that are redundant (i.e. that share the same information as another *k-mer*).

The noise threshold is determined empirically from the input samples and is calculated for each separate run of GECKO. To do this, we count the number of times a *k-mer* count appears in one sample with null values in all other samples from the same group for the same *k-mer*. Starting at a *k-mer* count of 1, we search how many times the value 1 appears for a *k-mer* in one sample with 0 in every other sample for the same *k-mer*. We then iterate this process for *k-mer* counts 2, 3, etc. When this frequency drops dramatically as determined by the slope of frequency counts (determined by calculating the derivative at each point), we consider that we are above background and set the threshold as the *k-mer* count just before the greatest inflection of the slope (Supplementary Fig. 7).

To determine uninformative *k-mers*, that is *k-mers* that do not vary across input samples, we first discretize the *k-mer* counts using a chi-square statistic that determines the minimum number of discrete intervals with minimum loss of class attribute interdependence^32^. This algorithm is unsupervised and determines the existence and number of separate levels in continuous data. If there are no clear categories, the discretization will output a vector of 1’s. Following this discretization, if there is not a minimum of 10% of samples with a different level, then this *k-mer* is considered uninformative. By default, this minimum number is set at 10% of the size of the input condition with the least replicates. For example, if the condition with the least replicates has 30 samples, then at least three samples must have a different discretized level to the other samples.

To eliminate redundant *k-mers* we use symmetric uncertainty (SU) between pairs of *k-mers*. Instead of comparing each *k-mer* to all other *k-mers*, we first split the *k-mers* into buckets of equal size and perform pairwise comparisons within a bucket. To determine which *k-mers* will be bucketed together, we calculate the sum of their counts across samples. *k-mers* with a similar sum across samples are put together; *k-mers* within a bucket have a higher chance of being redundant than if they were randomly bucketed. When all *k-mers* within buckets have been compared and redundant *k-mers* filtered, this process of bucketing by sum and filtering is repeated. This process of bucketing the *k-mers* by sum lead to 10 times faster filtering process on smaller samples and larger gains with larger matrices.

The SU between two *k-mers* *A* and *B* is given by the formula:

$${\mathrm{{SU}}}\left( {A,\,B} \right) = 2 \times \left( {\left( {H\left( A \right) + H\left( B \right) - H\left( {A,\,B} \right)} \right) \div \left( {H\left( A \right) + H\left( B \right)} \right)} \right]$$

where *H*(*A*) and *H*(*B*) are the entropies of the two *k- mers* along the samples and *H*(*A*, *B*) is the entropy of the combined *k-mer* counts A and B along the samples.

The Entropy is given by the formula:

$$H\left( A \right) = - \mathop {\sum }\limits_i^G Mi/N^\ast {\mathrm{{log2}}}(Mi/N)$$

where *G* is the total number of *k-mer* frequencies given by the discretization step, *Mi* is the number of samples at the given discretization level *N* is the total number of samples. In our analysis, we empirically set the limit of SU at 0.7, above which two *k-mers* were considered as redundant.

GECKO keeps a record of all *k-mers* eliminated due to redundancy along with the ID of the *k-mer* that caused it to be eliminated. Thus, when the genetic algorithm finds a solution, GECKO can provide all the redundant *k-mers* that would have provided a similar solution.

All code for the data preparation was implemented in C++.

### The adaptive genetic algorithm

The algorithm begins by splitting the input data into a training and test set. The test set is created by randomly selecting a number of samples from each input category. By default the number of samples selected is 1/6th of the category with the smallest amount of samples. The test set is used to establish a final test score that will have no impact on the genetic algorithm’s evolution but allows us to estimate how well GECKO performs on a given dataset.

*Training*: At each generation of the AG, all individuals are scored based on their ability to classify the input samples using a machine learning algorithm. In this study, the algorithm used was a Linear Support Vector Classification (LinSVC). This method combines excellent results on smalls datasets and unbalanced groups with a good generalization potential, for a small computational resource cost. LinSVC is implemented in GECKO via the Scikit-learn package^33^. GECKO can also be used with a random forest model or neural networks, however these have higher computational costs and require dedicated hardware to be implemented within reasonable time-frames.

To calculate the fitness score of an individual at each generation we randomly split up the training set into two. 2/3 of the training set becomes the inner training set and the remaining 1/3 becomes the inner test set. We contrast the inner test set, which is used to score individuals at each generation of the adaptive genetic algorithm with the test set which is not used to train the adaptive genetic algorithm but instead is used to estimate the performance of our model. The inner split on the training data is random and is performed five times. The score of each individual is an average of these five iterations trained on the inner training sets and tested on the inner test sets. This rotation of the training data avoids sample batch effect biases at each generation.

*Natural selection*: After testing the fitness of each individual of our population we delete individuals with lower fitness scores. By default, this is 30% of the population. We call this process natural selection.

We sort the individuals by ascending rank and then apply the following probabilistic rule:

$$P-{\mathrm{{value}}} = \alpha X + \beta$$

where *X* is the individual rank and the following conditions are satisfied:

$$\mathop {\sum }\limits_n^N P-{\mathrm{{value}}} = 1$$

$$\begin{array}{*{20}{c}} N \\ {P-{\mathrm{{value}}}} \end{array} = \frac{{\begin{array}{*{20}{c}} {N/2} \\ {P-{\mathrm{{value}}}} \end{array}}}{2}\begin{array}{*{20}{c}} N \\ {P-{\mathrm{{value}}}} \end{array}$$

where *α, β* are scalar values, *N* is the size of the population, and $$\begin{array}{*{20}{c}} N \\ {P-{\mathrm{{value}}}} \end{array}{\mathrm{{and}}} \begin{array}{*{20}{c}} {N/2} \\ {P-{\mathrm{{value}}}} \end{array}$$ are, respectively, the probability for the individual rank *N* and rank *N*/2 to be deleted.

*Mutation and crossing over rates*: GECKO makes use of three different types of Genetic Algorithm. These adapt the mutation and cross-over probabilities depending on the homogeneity and the performances of the population in order to converge faster and more accurately.

The three algorithms are:

- A simple adaptative genetic algorithm^34^. This algorithm has a fixed factor for individuals for which the fitness is inferior to the average and a decreasing linear function for the better performing half of individuals.
- Another improved adaptive genetic algorithm^35^ that, similar to the simple adaptive genetic algorithm, has a crossover probability fixed above the average fitness, but uses exponential instead of the linear function for fitness values below the average.
- An improved adaptive genetic algorithm^36^ that models the probabilities with two linear functions, with a breakpoint for the individuals that have a fitness equal to the average fitness.

We recommend using the last model as it shows better exploration and higher convergence rates for the kind of data used for GECKO. This approach aims to maintain the population’s diversity while protecting good individuals from modifications. The mutation and cross-over probabilities are decreased when the individual’s fitness is high compared to the average and increased if it is low. Similarly, the probabilities are decreased when the population is heterogeneous and increased when the population is homogeneous to favor exploration of novel solutions. These probabilities are modeled by two linear functions depending on whether the individual is above the average fitness of the population or below it and is given by the formula below.

$$Pm = \left\{ {\begin{array}{*{20}{c}} {\frac{{k_1\left( {f_{{\mathrm{{avg}}}} - f} \right) + k_2\left( {f - f_{{\mathrm{{min}}}}} \right)}}{{f_{{\mathrm{{avg}}}} - f_{{\mathrm{{min}}}}}},\,f \, < \,\, f_{{\mathrm{{avg}}}}} \\ {\frac{{k_2\left( {f_{{\mathrm{{max}}}} - f} \right) + k_3\left( {f - f_{{\mathrm{{avg}}}}} \right)}}{{f_{{\mathrm{{max}}}} - f_{{\mathrm{{avg}}}}}},\,f \ge f_{{\mathrm{{avg}}}}} \end{array}} \right.$$

Here *f* is the individual’s fitness, *f*_min_ is the fitness of the population’s worst individual, *f*_avg_ is the population’s average fitness and *f*_max_ is the fitness of the population’s best individual. *k*1 is the rate applied when *f* = *f*_min_, *k*2 when *f* = *f*_avg_, and *k*3 when *f* = *f*_max_.

*Stopping criteria*: By default, GECKO will run for an input number of generations. The user may however choose to make use of a stopping criteria that will stop the algorithm prematurely. The stopping criteria is checked after at least 5000 generations of the genetic algorithm. At this moment, the number of occurrences of each *k-mer* in the population is calculated across bins of 500 generations from the start of the algorithm to the current generation. The top 1% of most frequent *k-mers* in each bin are selected. We then estimate the difference in *k-mer* composition between the current bin and all previous ones using a Hamming distance. This distance measures the quantity of highest scoring *k-mers* that are changing across generations. When the slope of Hamming distance across generations drops below 1%, the stopping criteria is triggered.

*Adding Gaussian noise*: The user may add Gaussian noise to the model to prevent overfitting. The characteristics of this noise are determined for each *k-mer* separately. They are a mean of 0 and a standard deviation equal to the standard deviation of the *k-mer* in the training set. The user can modify the level of noise by changing noisefactor which multiplies the standard deviation by the input value. This noise is generated at each training of machine-learning model and for each individual.

*tSNE visualization*: t-SNE plots are generated using scikit-learn with the default parameters but initialization with PCA. This initialization option allows for better reproducibility of t-SNE graphs. Below is the corresponding command-line: manifold.TSNE (n_components = 2, init = ‘pca’, random_state = 0, perplexity = 30.0, early_exaggeration = 12.0, learning_rate = 200.0, n_iter = 1000, n_iter_without_progress = 300).

### Reporting summary

Further information on research design is available in the Nature Research Reporting Summary linked to this article.

## Supplementary information

Supplementary information

Reporting Summary

Description of Supplementary Data

Supplementary data 1

Supplementary data 2

## Data Availability

The data that support the findings of this study are available from NCBI Gene Expression Omnibus under the accession numbers GSE100467 and GSE58889; the Cancer Genome Atlas under the Pan-Gyn cohort name; the database of Genotypes and Phenotypes under the accession numbers phs000435.v2.p1 and phs001050.v1.p1 but restrictions apply to the availability of these data, which were used under license for the current study, and so are not publicly available. Data are however available by submitting a request to these repositories.

## Code availability

GECKO is available at https://github.com/RitchieLabIGH/GECKO under the CeCILL license.
