# Supplementary material for: GECKO is a genetic algorithm to classify and explore high throughput sequencing data
Source: Commun Biol. 2019 Jun 20;2:222. doi: 10.1038/s42003-019-0456-9 (PMC6586863; doi:10.1038/s42003-019-0456-9)
Supplement: Supplementary file 1 — Supplementary information [file 42003_2019_456_MOESM1_ESM.pdf]

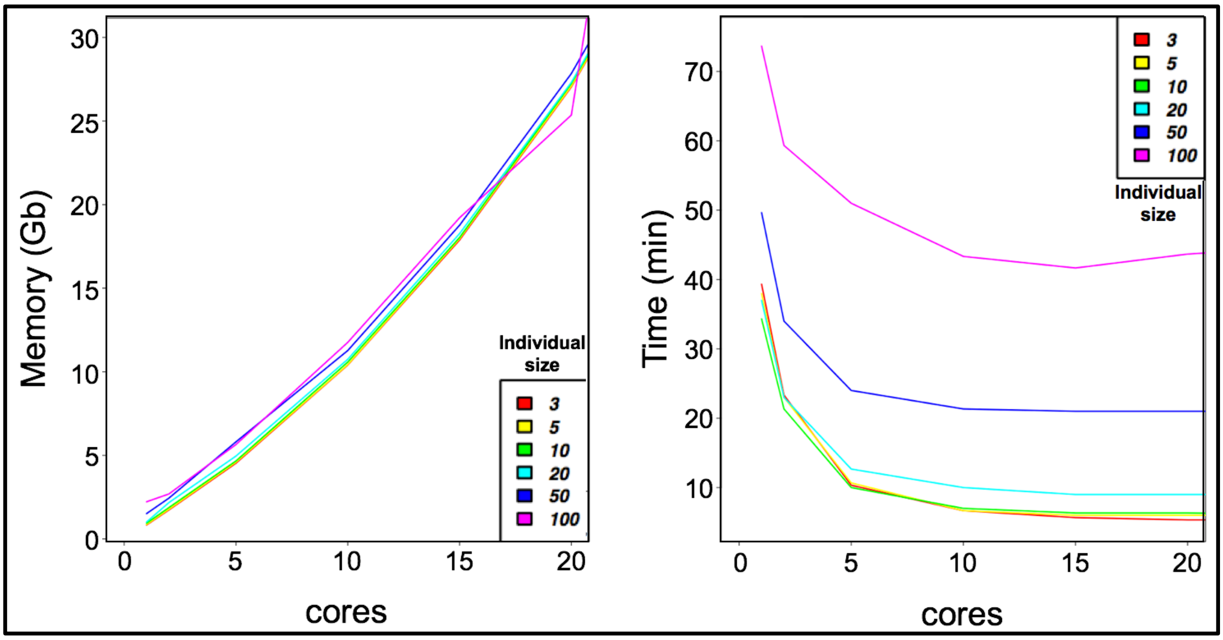

Supplementary Figure 1: Memory and time usage for 100 generations of the IAGA in GECKO for different numbers of k-mers per individual. The runtimes were performed in ideal conditions with no other users on the calculation node. The runtimes in the manuscript were recorded in real conditions with other users sharing the server.

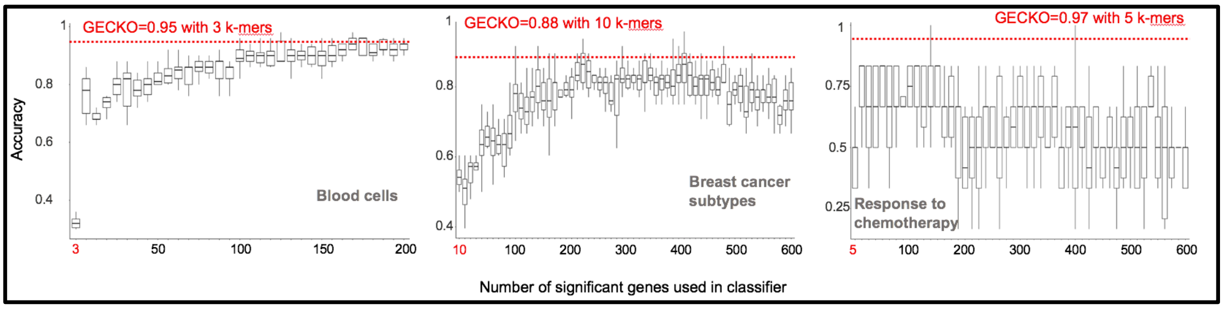

Supplementary Figure 2: comparison of classification accuracy between GECKO and classifiers based on an increasing number of differentially expressed genes

For each experiment in the manuscript, we first determine differentially expressed genes between conditions. We then use an increasing number of these genes to form a classifier (using the same SVM model as GECKO) starting with the genes that have the strongest p-values. For each number of genes used in the classifier, we ran the classifier 10 times using cross-validation and built a boxplot from these 10 replicates. These are compared to the median of 10 GECKO runs indicated by the red horizontal dashed bar.

microRNA levels were calculated using the nf-core smRNA-Seq pipeline v1.5 (github.com/nf-core/smrnaseq). Gene counts were downloaded from the TCGA website directly. We used DESeq2 to perform standard analysis as described in document "Analyzing RNA-seq data with DESeq2" that is available on Bioconductor website. For each dataset, we performed a one-versus-all analysis for each group. As described in[1] we took the genes with the best adjusted pvalues as the most differentially expressed genes.

1. Peng, L. *et al.* Large-scale RNA-Seq Transcriptome Analysis of 4043 Cancers and 548 Normal Tissue Controls across 12 TCGA Cancer Types. *Sci. Rep.* **5**, (2015).

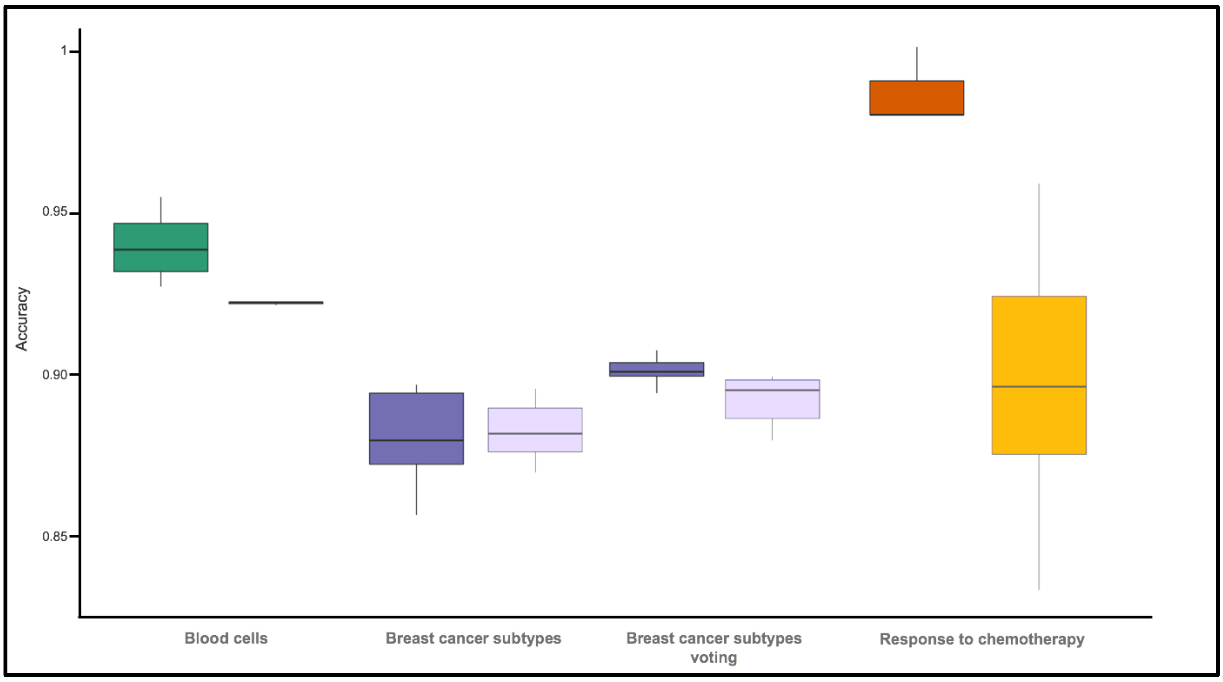

Supplementary Figure 3: comparison of classification accuracy between GECKO applied to k-mers (dark boxplots) and GECKO applied to transcript quantification values (light boxplots) from the same samples (n=10 separate runs)

GECKO was run 10 times for each experiment using either k-mers or FPKM values. microRNA levels were calculated using the nf-core smRNA-Seq pipeline v1.5 (github.com/nf-core/smrnaseq). Gene counts were downloaded from the TCGA website directly. For the breast cancer classification, we also added a voting mode to demonstrate that the k-mers had not been as extensively utilized as the FPKMs and thus adding a voting step increased the classification power for k-mers more than it did for FPKMs.

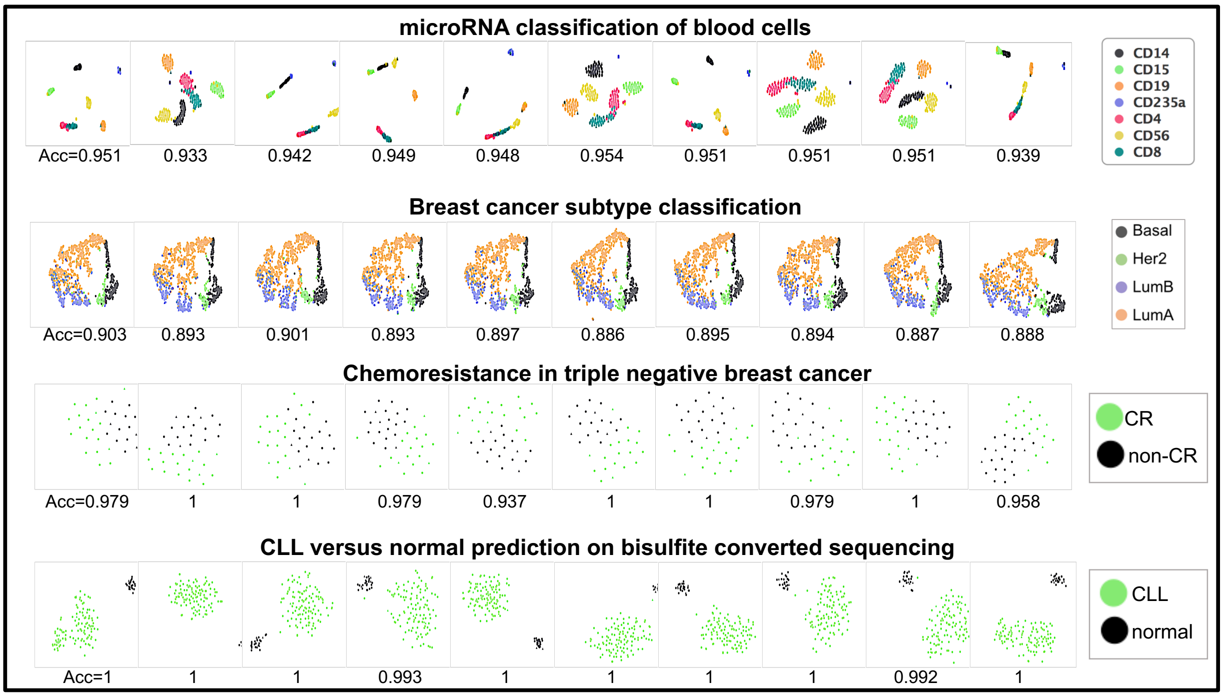

Supplementary Figure 4: classification accuracy and t-SNE separation for the 10 next best individuals in the same run of GECKO for all 4 experiments in the study in order of appearance in the GA.

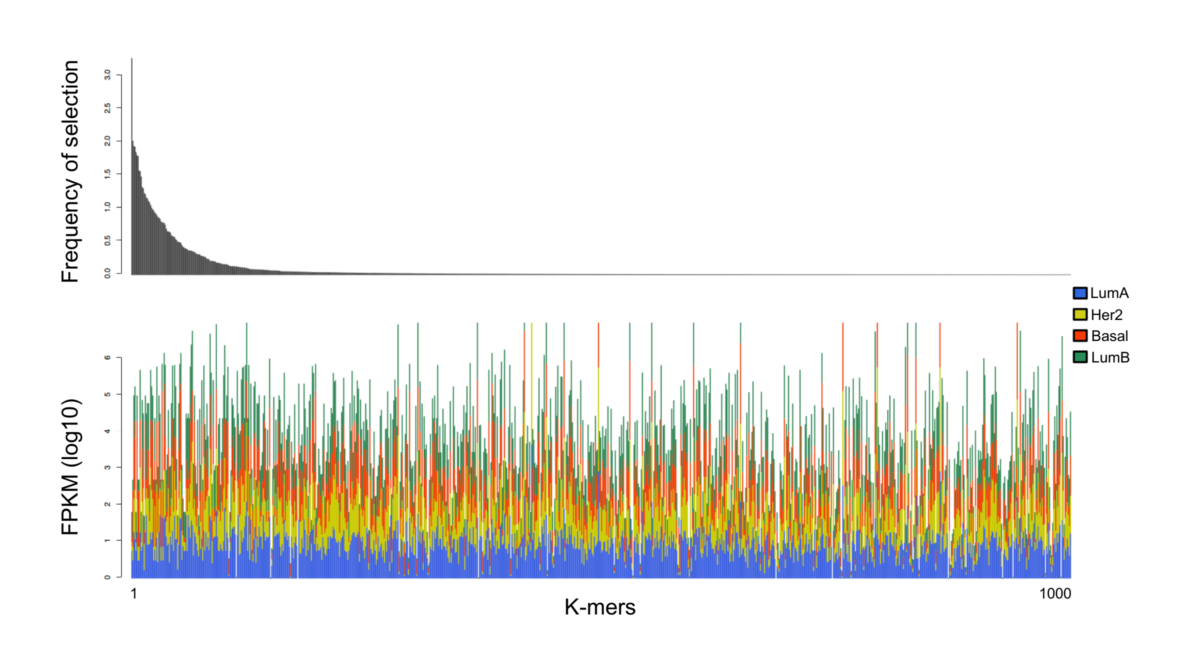

Supplementary Figure 5: gene expression relative to frequency of k-mer selection for the breast cancer subtype classification. For the 1000 most frequently selected k-mers, we compared the frequency with which they appeared during the genetic algorithm (as a percentage of total k-mer selections) with the FPKM values of transcripts to which they mapped. We aligned the k-mers with Blastn version 2.8.1 on the human transcriptome and the human genome from ensEMBL GRCh38. The parameters were evalue at 1000, gapopen at 5, gapextend at 2, penalty at 3 and word_size at 7.

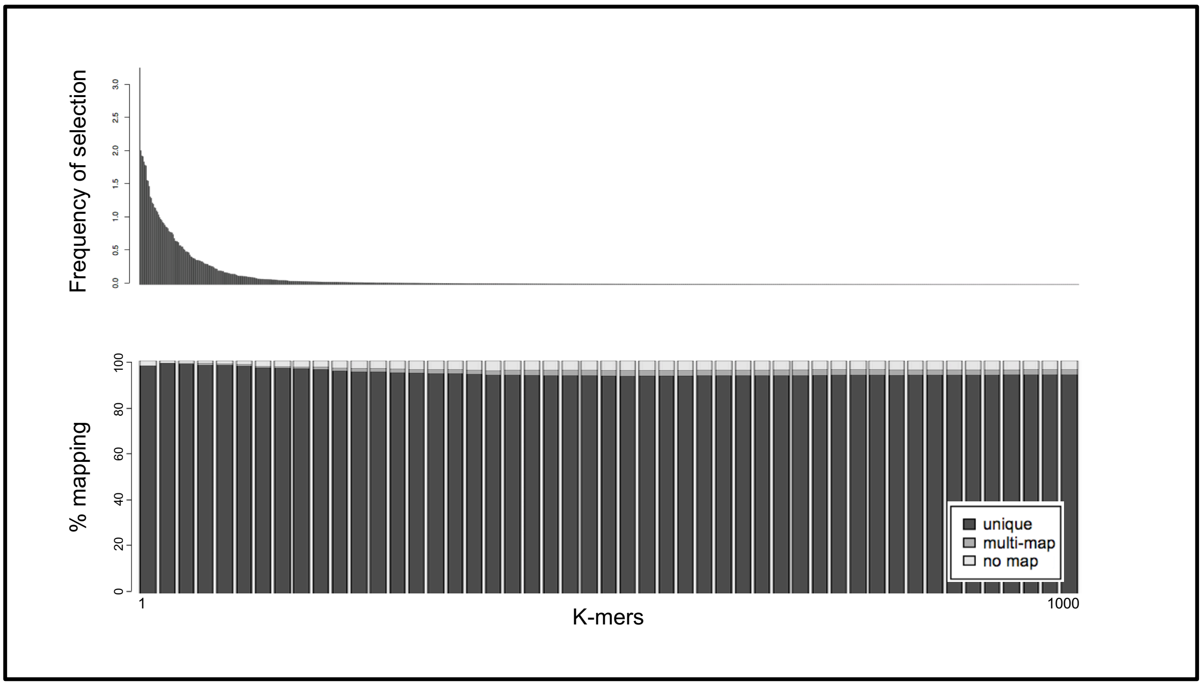

Supplementary Figure 6: number of mapping sites relative to frequency of k-mer selection for the breast cancer subtype classification. For the 1000 most frequently selected k-mers, we compared the frequency with which they appeared during the genetic algorithm (as a percentage of total k-mer selections) with the number of times they mapped to the human transcriptome or genome from ensEMBL GRCh38. The parameters were evalue at 1000, gapopen at 5, gapextend at 2, penalty at 3 and word_size at 7.

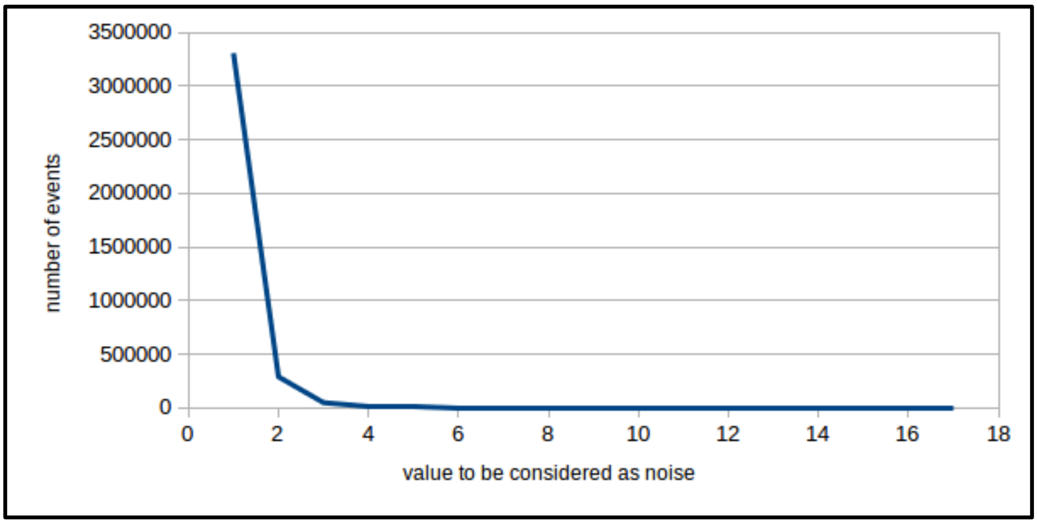

Supplementary Figure 7: noise threshold estimation. For each possible k-mer count in the matrix (x-axis), we count the number of k-mers for which this value appears in one sample but with the value 0 in all other replicates of the same experiment. In this example, the noise threshold would be set at 3.

Supplementary Table 1: GECKO parameters for experiments in the study

	MicroRNA	Beauty	TCGA	CLL
**Number of Generations**	12000	5000	20000	20000
**Number of Individuals**	600	840	600	840
**Kmers per individual**	3	5	10	20
**Elite**	2	1	1	2
**# Rotative test**	6	5	5	5
**Noise factor**	0	1	0	1
**Algorithm type**	IAGA	IAGA	IAGA	IAGA
**Kill ratio**	0,2	0,3	0,4	0,4
**Number of total k-mers after filtering**	85,569	27,794,147	91,609,675	2,516,986
**Number of possible combinations**	6,27E+14	4,16E+79	1,66E+37	1,04E+128

Supplementary Table 2: K mers used to classify breast cancer subtypes from the TCGA study

>kmer 1

GACTGTAGCAAGTTTTGACACACCCTCAAA

>kmer 2

CCATTCGCAGGGTTTCCAGGCCATTCTGAA

>kmer 3

ATAGAGTCATACAGTAGCTCAAAAGGCAAC

>kmer 4

CCGCAGCTCACAGCTGAGTTTAGCAGTGGA

>kmer 5

GACCCACCAGGGCTCCAGGCTGAAGCCTCA

>kmer 6

GCTGCTCCATCTCGCAGCGGAGCTGGGCCA

>kmer 7

TAGTCTGGGTTGCCTGTGTTTTGTGAGAGA

>kmer 8

ACCTGGACCGAGTGCTCATGGGCTACCAGA

>kmer 9

AGTAGCCTTATTTGCATATAGGGATTTAAG

>kmer 10

GAAAGCTTCTGAGGCATCTTGGCTATGTCC

Supplementary Table 3: K mers used to classify response to chemotherapy (BEAUTY)

>kmer1
CAAATCATTCAGAGATGGAGGTGGGAGGAG
>kmer2
AAGTTCGCTCATTTTCCTGTGCAATTTAAA
>kmer3
CAGAGATGGAGACGAAGGCCAGCATGGCTC
>kmer4
CGGAGGACAGCTCTGATGAGGAGGACATCC
>kmer5
CCCCCAGCCCACAGTGGTCTGGGCATCCCA

Supplementary Table 4: 20 k-mers from the winning individual from the CLL study

The k-mer size was set to 28 because sequences in the downloaded fastq file were of length 29 with a systematic error on the last nucleotide.

>kmer1
AAACAAAAACAAAAACGAAACCTAAACC
>kmer2
CGGTTTATGGATTGTTTTTAAGTTTTAA
>kmer3
AACACACCCTAAATCCAACGTCCTTTCC
>kmer4
CCTCTCCCCTCCCTACTTATTCAACCCA
>kmer5
CTCACCAATAACATCATAAACATCTCCA
>kmer6
CAAATAAAACTTAAATAATCAACCACCA
>kmer7
GTAATCCCTCCTCTACAAACTAAAACCA
>kmer8
CCTATTCCTATAAATACCTCTAAAACCG
>kmer9
CCCAATCGAAATCTACAAAAAAAAACCG
>kmer10
GGTTTTTGTTTTTGTTTTTGTTTTTTGA
>kmer11
AAACATATATAAATCACCTAACTTTCCA
>kmer12
ATTACCTCATAAATAACTACTATTACCC
>kmer13
AAAATTACAAACGCCCTCAACCACACCC

>kmer14
ATTTTCATCTCCATCCGCGAAACTCCCA
>kmer15
CCTTATAATCTTTACTCCACCAACTCCC
>kmer16
AATCCATCATTTAAAAATATAACTTCCA
>kmer17
AAACTTCCTTATCTACTCTACATTACCG
>kmer18
CCACAAAAAAAAAATTTAAAATAACCCC
>kmer19
GGGATATGTTTGTAGATGGAGGAGGAGA
>kmer20
CTTCCTAAAAATTAACTAATAAAAACCA

Supplementary Table 5: 3 k-mers most frequently used by winning organisms in the CLL study
These are bisulfite converted sequences and were mapped to “bisulfite converted” genomes generated by bismark to find their position.

>90528
AAACCCCCACTATTCCAACAAAAAATCC

>3539
ATCTCATAATACAATATTTTTTAAACCA

>107977
AATCCATCATTTAAAAATATAACTTCCA
