# Supplementary material for: GECKO is a genetic algorithm to classify and explore high throughput sequencing data
Source: Commun Biol. 2019 Jun 20;2:222. doi: 10.1038/s42003-019-0456-9 (PMC6586863; doi:10.1038/s42003-019-0456-9)
Supplement: Supplementary file 3 — Description of Supplementary Data [file 42003_2019_456_MOESM3_ESM.pdf]

Description of Additional Supplementary Files

File Name: Supplementary Data 1
Description: Data used to create Figure 2b

File Name: Supplementary Data 2
Description: Data used to create Figure 2c
